# Student mistakes and teacher reactions in bedside teaching

**DOI:** 10.1007/s10459-023-10233-y

**Published:** 2023-05-11

**Authors:** Hannah P. K. Rubisch, Anna-Lena Blaschke, Pascal O. Berberat, Cornelia S. Fuetterer, Bernhard Haller, Martin Gartmeier

**Affiliations:** 1 Lehrstuhl für Medizindidaktik, medizinische Lehrentwicklung und Bildungsforschung Technical University of Munich (TUM), TUM School of Medicine, TUM Medical Education Center, Ismaninger Straße 22, 81675 Munich, Germany; 2 Institute of AI and Informatics in Medicine, Technical University of Munich (TUM), TUM School of Medicine, Munich, Germany

**Keywords:** Bedside teaching, Medical education, Student mistakes, Teacher feedback, Teaching methods, Teacher reaction, Video study

## Abstract

We analyse interactions between teachers and students during video-recorded bedside teaching sessions in internal medicine, orthopaedics and neurology. Multiple raters used a high-inference categorical scheme on 36 sessions. Our research questions concern the types of student mistakes, clinical teachers’ reactions to them and if they use different strategies to address different types of mistakes. We used a Poisson model and generalized mixed models to analyse these research questions. Most frequently, students made reproduction mistakes. Relatively high rates of rejection and a similar prevalence of low and high levels of elaboration and correction time for students were observed. Reproduction mistakes were associated with the highest level of rejection and the lowest level of elaboration. High levels of elaboration were observed when students were applying skills in new situations. Students were most often allowed time to correct when mistakes in the areas of analysis or application of skills and knowledge had occurred. There is a decrease in the rate of making mistakes for neurology and orthopaedics compared to internal medicine. Reproduction mistakes influence significantly the outcome feedback compared to application mistakes. Analytic and reproduction mistakes influence elaboration significantly compared to application mistakes. We found a significant effect whether the lecturer allows time for correction of reproduction mistakes compared to application mistakes. These results contribute to the understanding of interactive, patient-centred clinical teaching as well as student mistakes and how teachers are reacting to them. Our descriptive findings provide an empirical basis for clinical teachers to react to student mistakes in didactically fruitful ways.

## Introduction

Bedside teaching is a long-standing core element of most medical curricula and a *signature pedagogy* (Shulman, 2005) of medical education. Nevertheless, the amount of time dedicated to bedside teaching has significantly declined over the past years (Ahmed, 2002). With the coronavirus pandemic starting in 2019 (COVID-19), the time physically spent at bedside further decreased (Miller, 2020). This accentuates the need to use the time dedicated to bedside teaching effectively. This challenges medical education research to determine which factors are essential for the quality of bedside teaching. In this context, one aspect which has not yet been analysed systematically is how clinical teachers handle student mistakes (Fischer et al., 2006; Lester & Tritter, 2001) in didactically fruitful, learning-oriented ways. In being a practice- and discussion-oriented instructional format, bedside teaching offers many opportunities for students to actively contribute (e.g. through engaging in clinical examination, clinical reasoning and decision-making) (Burch et al., 2006; Celenza & Rogers, 2006; Dreiling et al., 2017; Gonzalo et al., 2013; Nair et al., 1998; Peters & Cate, 2014; Ramani, 2003; Williams et al., 2008). In performing these activities, students also make mistakes, which represent valuable opportunities to reflect and learn. As argued by Fischer et al., 2006; Tulis, 2013, whether mistakes in instructional settings are learned from depends upon (clinical) teachers’ reactions to mistakes. Substantial research has shown the importance of whether teachers manage to create a constructive and emotionally safe atmosphere in which student mistakes can be openly addressed and discussed in ways that allow to identify and correct underlying misconceptions (Edmondson, 1999; Tsuei et al., 2019).

Drawing upon these points, we empirically investigate which types of student mistakes occur in bedside teaching, how clinical teachers react to these mistakes and to which degree they adapt their reactions to different types of student mistakes. We video-recorded a sample of bedside sessions from various clinical domains, identified student mistakes in these sessions and rated the subsequent teacher reactions using a theory-based categorical scheme. We will also characterise bedside teaching as an instructional method, elucidate the importance of learning from mistakes and the role of clinical teachers in this context.

### Bedside teaching in medical education

Bedside teaching can be briefly characterised as ‘the process of active learning in the presence of a patient’ (Nair et al., 1998, p. 159). Weinholtz et al. stated that ‘according to medical students systematic self-reports’, the learning in the presence of patients, is ‘among the most influential learning experiences encountered in medical school’ (Coppernoll & Davies, 1974; Weinholtz et al., 1989).

In actual clinical teaching, bedside teaching is practised quite heterogeneously (Peters & Cate, 2014). Mostly, bedside teaching for undergraduate students takes place in small groups led by a doctor who is temporarily released from ward work (Raupach et al., 2009). Its primary purpose is to educate the students and less to care for the patients, as they are not directly cared for by the respective students (Dybowski & Harendza, 2013). Mostly, students are not passive recipients of information in bedside teaching, but are engaged in active and skills-/knowledge-oriented forms of learning. These involve, among others, clinical communication, examination techniques, clinical reasoning and case presentation skills. These skills are practised along with various typical phases of bedside teaching in which students assume responsibility for preparing a patient case and interviewing and/or examining the patient (Celenza & Rogers, 2006; Dreiling et al., 2017; Gonzalo et al., 2013; Nair et al., 1998; Peters & Cate, 2014; Williams et al., 2008). The point of these didactically orchestrated activities is to help students to learn how to correctly apply their knowledge and skills to specific patient cases in professional and competent ways. Furthermore, bedside teaching gives students many valuable opportunities to make mistakes and learn from them.

### Definition and types of mistakes

To substantiate the latter point, we will define mistakes and introduce a taxonomy of students’ mistakes applied in the present study. Moreover, we will elaborate on how learning from mistakes in the context of bedside teaching can be conceptualised in relation to the different types of mistakes.

The term *mistake* is used to describe individual actions or their outcomes. Labelling these as mistakes means that an action (or its outcomes) deviates in a deficient way from a specific goal or standard, which is relevant in the respective context (Bauer, 2008). In medicine, in many cases, expert professionals are responsible for performing such judgements. Thus, for the purpose of the present study in the context of bedside teaching, we define mistakes as situations wherein a clinical teacher explicitly judges a (verbal or practical) student contribution as incorrect.

Drawing upon this definition, we differentiated several types of mistakes on the basis of the classification advanced by Anderson and Krathwohl (2001) based on Bloom’s well-established taxonomy (1956).^1^ This hierarchical model describes how knowledge is used to perform cognitive operations in different levels of complexity, from recalling knowledge, demonstrating the understanding of knowledge, applying knowledge to solve problems and, finally, using knowledge to perform differential analyses of subject matter.^2^ On this basis, we differentiate student mistakes along with different types of knowledge (e.g. facts, concepts and procedures) and the underlying levels of cognitive processing (cf. Table 1, Mindnich et al., 2008).

**Table 1:** Categories of student mistakes

Main category (Reference to Anderson et al., (2001))	Brief description
Reproduction mistakes *(Remembering)*	Misconceptions regarding basic biomedical subject matter
Comprehension mistakes *(Understanding)*	Wrongful connections or conclusions from basic biomedical subject matter
Wrong application of skills *(Applying)*	Incorrect application of skills
Wrong application of knowledge *(Applying)*	Incorrect application of knowledge
Analytic mistakes *(Analysis)*	Mistakes in the areas of analysis and/or evaluation of medical problems or creation of diagnostic and/or therapeutic strategies
Other mistakes	Mistakes that do not belong into any of the above groups
Mistakes related to misunderstandings/misinterpretations	Teacher instruction being vague or incomplete or correct student reaction to misjudged teacher initiative due to misunderstanding/misinterpretation Wuttke (2008, p. 97)

*Remembering information* is the basis of Anderson and Krathwohl’s taxonomy. Mistakes relating to this level are *reproduction mistakes*. Such mistakes occur when students wrongfully remember a concept that they have previously learned, e.g. because they mix up approaches or have a wrong/incomplete understanding of the subject matter (Olde Bekkink et al., 2016). An example of a reproduction mistake is when the medical teacher (MT) discussed important anamnestic questions with the students (S1 and S2) during tumour anamnesis. *MT: Then the B symptoms are important. And what do we mean by that?*

S1: Fever

MT: Exactly, there are three symptoms?

S1: Fatigue

S2: Night sweats, weight loss

MT: Fatigue fits in but is not part of the definition.

V1F1^3^

On the next level, *understanding*, we speak of *comprehension mistakes* when a student has not properly understood a specific concept or process. The following situation shows a comprehension mistake. It happened when the MT discussed about a patient with suspected diffuse large B-cell lymphoma to practise clinical reasoning with the students.

MT: This CRP increase may have also been caused by the tumour. How can you differentiate between an increase due to the tumour and an increase because of an infection with bacteria?

S: We can determine the IL-6 (Interleukin 6) value.

MT: IL-6 is a good marker as it increases rapidly after infection, but it is an inflammatory mediator like CRP.

You can determine the Procalcitonin value which may indicate a bacterial infection if positive.

V3F3

Then, we differentiate between two forms of mistakes occurring in the field of *application*, specifically in the areas of *skills* and *knowledge*. Skills-related application mistakes occur when a student performs an examination on a patient wrongfully.

S1 does not follow the predetermined sequence when conducting the abdominal examination

MT: The technique of percussion was good, however is there a correct sequence which makes more sense than what you just demonstrated?

S1: … [Student pauses]

MT: If we suspect ascites, how would it make the most sense?

S2: I would go from the centre to the side.

MT: yes exactly, quite right

V1F3

Similarly, students may also apply knowledge in wrongful ways. *Application mistakes* regarding knowledge may occur when a student solves problems formulated by the lecturer. In the following example, the setting is the sonography of the abdomen with colour duplex:

MT: What do the colours show me?

S1: Red means it is flowing towards the transducer, blue is flowing away.

MT: How does the blood flow in the portal vein?

S2: Towards the liver, that is away from the transducer.

MT: No, towards the transducer, you can see that quite well here.

V1F7

Relating to the level of *analysis*, we further differentiate *analytic mistakes*. Such mistakes typically occur in the context of higher-order mental processes, such as clinical reasoning, and are characterised by wrongful analysis or evaluation of the subject matter. The following example is about secondary prophylaxis after a stroke:

MT: Duplex sonography shows plaques in the medial cerebral artery. Which medication do we give the patient?

S: ASA 100 mg

MT: Yes, that's right. Let’s say many plaques are detected.

S: DOACs

MT: Would you give him an ASA 100 mg and a DOAC now?

S: Yes

MT: All of you?

Ss: [laughter]

MT: If I ask so stupidly. When do you give DOAC or Marcumar? For atrial fibrillation. If the patient doesn't have atrial fibrillation, we don't need to give him anticoagulation.

V16F1

Finally, we used two more categories of mistakes not related to Anderson and Krathwohl’s scheme: some student mistakes relate to the misunderstandings or misinterpretations of the teacher’s questions. Such mistakes sometimes occur when the teacher’s instructions are vague/incomplete or when the clinical teacher misjudges a students’ reaction (Wuttke, 2008). Finally, we used one residual category, that is, *other mistakes*, for instances of mistakes that did not fit into any of the above categories.

### Teacher reactions to student mistakes in bedside teaching

‘As medical students, whether preclinical or on the wards, we live in fear, afraid to make a mistake, to forget a fact, to appear stupid in front of peers or superiors […].’ Miller describes (2010, p. 1629). Bedside teaching is effective as a didactic format because clinical teachers can constructively address and discuss students’ misconceptions (which manifest in the mistakes students make). Nevertheless, it is not trivial for such learning to occur. In a bedside teaching study among fourth-year medical students Olasoji described that ‘a clinical teacher belittles and/or humiliates a student who has fallen short of expected performance’ (2018, p. 483). As existing theory (Bauer, 2008; Tulis et al., 2016; Weingardt, 2004) and empirical evidence (Metcalfe, 2017; Spychiger et al., 1998; Tulis, 2013) show, such behaviour can result in students feeling anxious and uncomfortable—emotions that impair learning in general and mistake-related learning in particular. Therefore, clinical teachers should strive to create an atmosphere in which students feel psychologically safe (Edmondson, 1999; Newman et al., 2017). Such an atmosphere is considered as a prerequisite for students’ willingness to verbalise their assumptions and thoughts behind an erroneous contribution or action, which, in turn, is a prerequisite for addressing the mistake constructively. As bedside teaching is a very interactive small-group format, it offers favourable conditions for such learning. Nevertheless, we assume that the direct reactions clinical teachers show when student mistakes occur are decisive regarding whether an error-friendly climate is created.

In the present study, we operationalised teacher reactions drawing upon three categories adopted from Wuttke (2005, 2008) and Crespo (2002), i.e. *feedback*, *elaboration* and *time for correction* (cf. Table 2). Regarding feedback, we differentiate between the *explicit rejection* of a student statement or an action and *further inquiry*. An example for the former would be a teacher responding *‘No, that’s wrong!’* to a student statement. Examples for further inquiry are questions like: *‘Are there any other ideas?’* or *‘What do the others think about this?’*.^4^ Then, we coded the degree of elaboration after a student mistake. *Low elaboration* was coded if only a brief correction without any further discussion was undertaken. In contrast, we coded *high elaboration* in cases where clinical teachers initiated discussion and reflection. Finally, we coded whether teachers allowed ample *time for students to correct the mistakes* they had made. We coded *time* when the students were given time to reflect their answer; when comprehension questions or student utterances demonstrating contemplation, such as *‘Ah yes, I understand’* were occurring; or when students were correcting their mistakes themselves.

**Table 2:** Categories of teacher reactions to student mistakes

Teacher reactions to student mistakes/procedure of correction		
Feedback	Explicit rejection	The teacher/another student directly rejects or immediately corrects a statement or an action (e.g. a false examination technique)
	Further inquiry	Wrong statements or examination techniques are not immediately corrected. Instead, the student group is asked for their judgement
Level of elaboration	Low	The teacher does not further discuss wrongful assumptions or reasons behind a mistake
	High	The teacher initiates the discussion and/or elaborates about the mistake (for qualitative analysis of feedback cf. Crespo (2002) and Wuttke (2005, 2008))
Time for correction of a mistake^*^	No	The teacher does not leave a student sufficient time to self-correct a mistake
	Yes	The student is given time to correct a mistake. The student’s process of contemplation may be recognised by expressions, such as ‘*ah yes, I understand*’, further questions or self-correction of the mistake

*Adequacy of time span assessed, absolute time negligible (Wuttke (2008))

One research question in this study aims to determine whether clinical teachers adapt their reactions to the different types of student mistakes in didactically fruitful ways.

Types of mistakes and teachers’ responses are both contextual and can vary with the learning situation itself. Depending on learning content and subject, different forms of bedside teaching are described (Aldeen & Gisondi, 2006; Almutar et al., 2013; Peters & Cate, 2014). Thus, it is useful to distinguish between different medical disciplines in the analysis.

Hence, in the present study, we address three research questions:

1. Which types of student mistakes occur during bedside teaching lessons?

2. How do clinical teachers react to the different types of student mistakes in bedside teaching lessons?

3. Do clinical teachers use different strategies to address different types of student mistakes?

Moreover, we aim to investigate how clinical teachers in three medical disciplines, i.e. internal medicine, neurology and orthopaedics, handle student mistakes in bedside teaching and then analyse the research questions comparing the individual medical disciplines.

## Materials and methods

### Ethical considerations

The ethics committee of the TUM Rechts der Isar University Hospital reviewed and approved the present study (Application code 360/18 S). We informed all teachers and students about the study well in advance of the course via email. We only filmed lessons when the respective teachers and all of the students had declared their willingness to be part of the study. The students in the courses selected for video recording were given the opportunity to be assigned to another course if they did not wish to be part of the study (seven students used this opportunity). At the beginning of each lesson (before starting the video recording), a member from our research team was present to again inform the participants about the study, respond to open questions and collect written consent from all participants. We started the video recordings only after all the individuals in the room had declared consent. All of the patients involved in the present study had also declared verbal consent before the bedside lesson, and their written approval was obtained immediately afterwards.

### Sample

In a preliminary study, we had videotaped three clinical bedside teaching lessons in the summer of 2018. Such data were used to examine and optimise the data collection procedure, especially the coding scheme used in the main study. Therein, 36 bedside lessons were filmed (12 internal medicine, 12 neurology and 12 orthopaedics) in the winter semester of 2018–2019. All analyses reported in this paper rely on data from these lessons. The 36 lessons were given by 24 different teachers. This means we filmed 12 teachers once and 12 teachers twice. The demographic data of teachers and students (teachers: medical discipline, age and sex; students: age, sex and year of medical studies [cf. Blaschke et al. (2022)]), were collected by means of questionnaires handed out at the end of the lessons. The lecturers in the present sample had an average age of 33 years (range 26–46 years). Six teachers were female, and 18 were male. A total of 259 students took part in the videotaped bedside lessons and were visible in the videos (N_STU_V_ = 259). Out of these, only 245 of these students returned the distributed questionnaire (94.6% return rate, N_STU_Q_ = 245) which means we can only provide demographic information on this part of our sample. 137 (58.5%) out of these 245 respondents were female, 97 (41.5%) were male and 11 (4.5%) did not specify their gender. On average, the bedside teaching lessons were attended by seven students (*SD* = 2.7, *Min* = 4, *Max* = 13) which mostly studied in their second clinical year (fourth year overall). Their median age was 23 years (IQR 22–24 years, mean age 23.7 years (*SD* = 3.1)).

Our sample comprised three general subject areas to which the seminars were associated to, i.e., internal medicine (with subdisciplines: gastroenterology, haemato-oncology, cardiology & pneumology and rheumatology, endocrinology & nephrology), neurology and orthopaedics.

### Video recording of bedside teaching lessons

**Fig. 1:**
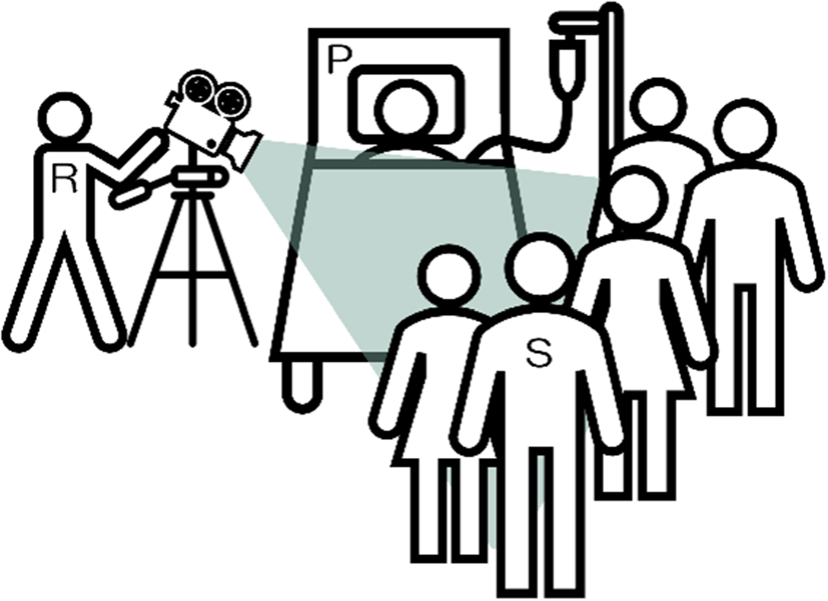
Camera setup in the patient room (components of this graphic are provided unrestricted and copyright-free by Microsoft, figure adapted from Blaschke et al. (2022)). P = Patient, R = Researcher, S = Student

Different methodological approaches have been introduced to investigate bedside teaching, among them being audiotaping (Al-Swailmi et al., 2016), using structured questionnaires (Ben Salah et al., 2015) and video-analytic research methods (Rees et al., 2013; Rizan et al., 2014; Weinholtz et al., 1986). As it allows for fine-grained analyses of how teachers and students interact, we argue that videography is particularly promising (Janik & Seidel, 2009; Seidel, 2005; Seidel & Thiel, 2017). The video recordings were made by trained staff from our research team using one camera (cf. Figure 1). This camera followed the group when they changed location (e.g. from a meeting room into a patient room). In the patient room, we made sure that the faces of the patients were not visible on the videos to retain their anonymity. To do so, the cameraperson stood next to the head of the patient’s bed, and the camera was directed towards the students. In this way, the patients were not visible but audible in the video recordings.

### Categorical scheme and coding process

To analyse the video material, a hierarchical categorical scheme was created on the basis of the published rating schemes used in previous video studies (Seidel, 2005). In the first step, all mistakes were marked along with other codes. In a succeeding round, the mistakes were approved and classified more precisely using the codes already described in the theoretical background of the study (cf. Tables 1 and 2 for code descriptions). Our basis for assigning the code *student mistake* was that a correction was made by a clinical teacher (cf. Mindnich et al., 2008) and included mistakes on both verbal and nonverbal levels (e.g. mistakes occurring during a clinical examination). A Cohen’s kappa of 0.84 was achieved for the classification of a mistake. To classify the mistakes of our sample more precisely in the next step, we first used the video material from the preliminary study to train the raters. After a satisfactory agreement was reached, we moved on to the main study. In the present study, a Cohen’s kappa of 0.84 for the categorical scheme *type of the student mistake*, 0.64 for *feedback*, 0.91 for the *level of elaboration*, and 0.66 for *time for correction* were achieved.

### Statistical analyses

We used *Microsoft Excel* and *Mangold Interact* to code and *SPSS* (*IBM SPSS Statistics*, Version 28.0. Armonk, NY) and *R* 4.0.1 (*R Foundation for Statistical Computing*, Vienna, Austria) to perform the calculations. With groups of equal size (3 times 12 seminars) and non-normally distributed frequencies of mistakes, we used the Kruskal–Wallis test to detect the differences in central tendency. As a post-hoc test, we applied the Bonferroni test and thereby correct for multiple testing in the individual test. We modelled the data according to the student mistakes per hour and used Poisson models for the mistake rate, adjusting for the medical discipline. To investigate the effect of the different disciplines to the clinical teacher reactions we provide generalized mixed models. In the following section, our data are presented by absolute and relative frequencies. Stacked bar charts are used to display the distribution of the relative frequencies of the categorised teacher reactions to student mistakes within bedside teaching lessons.

## Results

We observed substantial differences regarding the length of the filmed lessons. We found a mean duration of 128.9 min (*SD* = 30.5 min); the shortest lesson lasted 79.9 min and the longest 181.9 min, with an IQR of 57.0 min from 103.6 to 160.6 min. Regarding the three main medical disciplines, we found that lessons were shortest in orthopaedics with an average duration of 116.1 min (*SD* = 29.5 min) and longest in internal medicine with an average duration of 144.3 min (*SD* = 22.7 min) per lesson. For neurology, we observed an average duration of bedside lessons of 126.4 min (*SD* = 33.5 min).

Most student mistakes occurred in internal medicine (199) and in neurology (123). In orthopaedics, the least number of mistakes was observed (50). In sum, we observed 372 mistakes; this equals 10.3 mistakes per bedside teaching session on average. At the individual bedside teaching level, this translates to a mean of 6.4 mistakes per hour in internal medicine (*SD* = 5.2), 4.5 mistakes per hour in neurology (*SD* = 3.1) and 2.1 mistakes per hour in orthopaedics (*SD* = 1.5). The difference in central tendency in the frequency for the individual seminars between internal medicine and orthopaedics was statistically significant (*p* = 0.012 after Bonferroni correction).

**Fig. 2:**
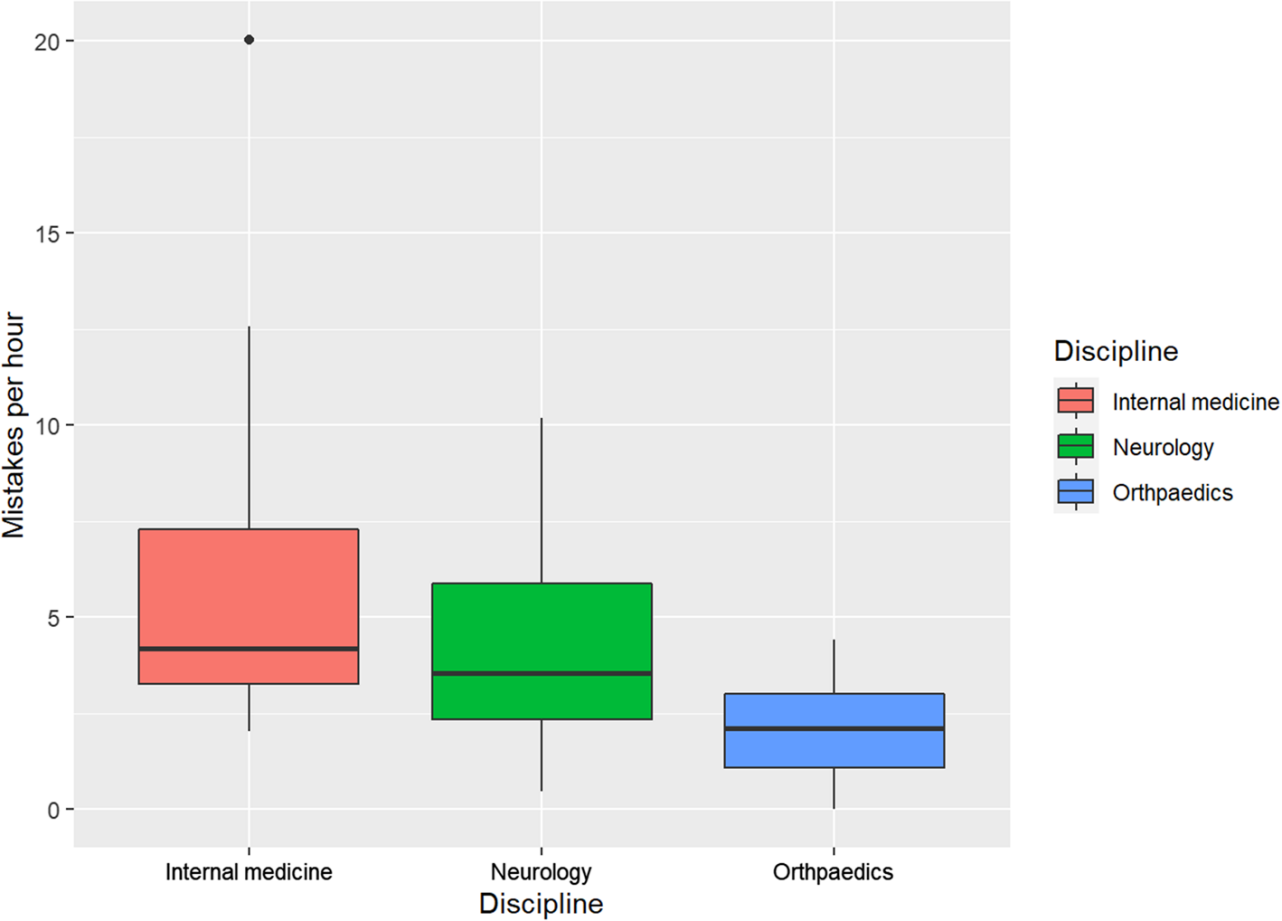
Proportion of total mistakes of each discipline per total video duration of the according discipline

We looked at the proportion of total mistakes of each discipline per total video duration of the according discipline (Sum of events/Sum of total time, cf. Figure 2). In case of internal medicine the proportion was directly 0.0019, for neurology it was 0.0014 and for orthopaedics it was 0.00060.

The ratio of the mean mistakes per hours of neurology to internal medicine was directly obtained by 0.0014/0.0019 = 0.71 (cf. Table 3), equalling the same value as in the Poisson model (0.71). Furthermore, the mistake rate of orthopaedics compared to internal medicine was: 0.00060/0.0019 = 0.31 as the corresponding coefficient estimate of the Poisson regression.

**Table 3:** Ratio of the mean of mistakes per hours of the according discipline

	Discipline		
Proportion	Internal medicine	Neurology	Orthopaedics
	0.001915229	0.7057836	0.3124473

### Poisson models results

Table 4 shows a Poisson model of the mistake rate per discipline. We modelled mistakes per hour as outcome in dependence of the covariate “Discipline”, with the intercept indicating the log mistake rate for internal medicine. Exp(− 6.3) = 0.0019 was the predicted mean number of mistakes for the discipline internal medicine. The rate ratio of neurology as compared to internal medicine was exp(Neurology) = exp(− 0.35) = 0.71. This means that there was a decrease in the rate of making mistakes for the discipline neurology compared to internal medicine. The rate ratio of orthopaedics also indicated a decrease in orthopaedics compared to internal medicine by exp(− 1.2) = 0.31.

**Table 4:** Poisson model modelling the mistake rate in dependence of the discipline

Poisson model for the mistake rate in dependence of the discipline						
*Deviance residuals*						
Min	1Q		Median	3Q		Max
− 3.7504	− 2.3734		− 0.5542	0.6415		6.9002
*Coefficients*						
	Estimate		Std. Error	z-value		Pr (> ǀzǀ)
(Intercept)	− 6.25792		0.07089	− 88.279		< 2e-16 ***
Discipline neurology	− 0.34845		0.11470	− 3.038		0.00238 **
Discipline orthopaedics	− 1.16332		0.15819	− 7.354		1.93e-13 ***
–						
Signif. codes	0 ‘***’	0.001 ‘**’	0.01 ‘*’	0.05 ‘.’	0.1. ‘’	1
(Dispersion parameter for poisson family taken to be 1)						
Null deviance: 252.59 on 35 degrees of freedom						
Residual deviance: 186.66 on 33 degrees of freedom						
AIC: 321.79						
Number of Fisher Scoring iterations: 5						

**Fig. 3:**
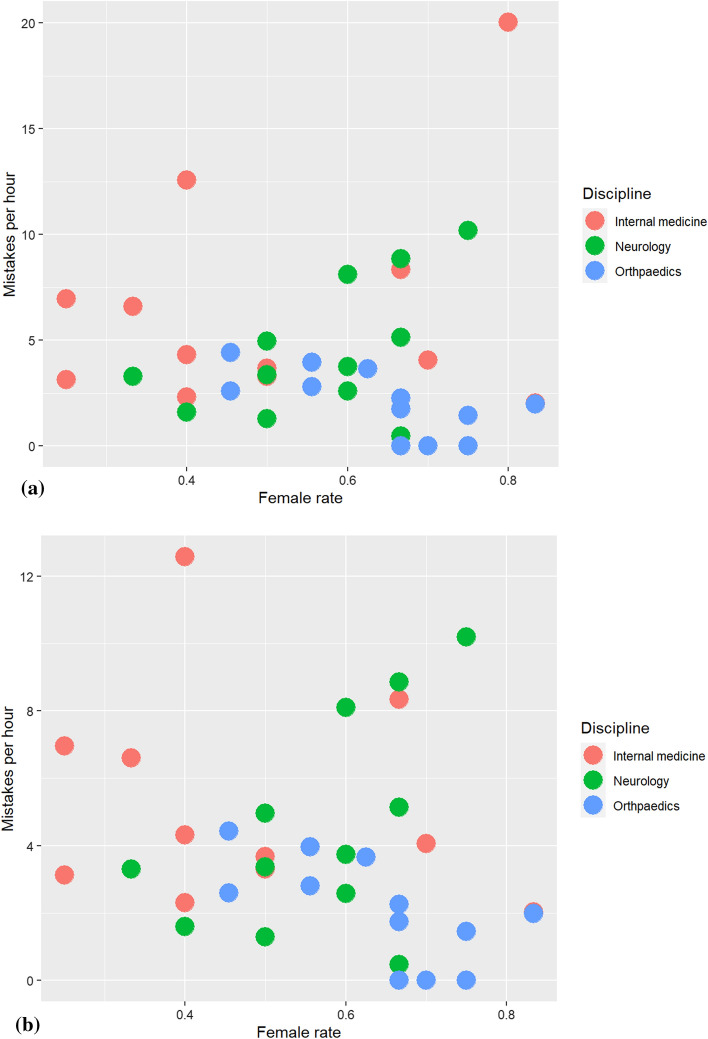
**a** Mistakes per hour of each discipline plotted against covariate female rate, **b** Mistakes per hour of each discipline plotted against covariate female rate without outlier

We provide a model for the mistake rate, adjusting for the discipline as well as the number of students (Table 5) and the rate for female students in the seminar (Table 6a). Figures 3a and b explain why the female rate appeared significantly in Table 6a: As expected, fitting the model again without the outlier of 20 mistakes per hour (internal medicine) removed the significant effect of the covariate female rate (cf. Table 6b).

**Table 5:** Poisson model for the mistake rate, adjusted for the discipline, as well as the number of students

Poisson model for the mistake rate adjusted for the discipline and the number of students						
*Deviance residuals*						
Min	1Q		Median	3Q		Max
− 3.6736	− 2.2104		− 0.3507	0.3518		6.7597
*Coefficients*						
	Estimate		Std. Error	z-value		Pr (> ǀzǀ)
(Intercept)	− 5.82527		0.21636	− 26.924		< 2e-16 ***
Discipline neurology	-0.32336		0.11592	− 2.789		0.00528 **
Discipline orthopaedics	− 0.75181		0.25660	− 2.930		0.00339 **
Number of students	− 0.08145		0.03943	− 2.065		0.03888 *
–						
Signif. codes	0 ‘***’	0.001 ‘**’	0.01 ‘*’	0.05 ‘.’	0.1. ‘’	1
(Dispersion parameter for poisson family taken to be 1)						
Null deviance: 252.59 on 35 degrees of freedom						
Residual deviance: 181.84 on 32 degrees of freedom						
AIC: 318.98						
Number of Fisher Scoring iterations: 5

**Table 6a.** Poisson model for the mistake rate, adjusted for the discipline, as well as the number of students and the rate for females

Poisson model for the mistake rate adjusted for the discipline as well as the number of students and the rate for females						
*Deviance residuals*						
Min	1Q		Median	3Q		Max
− 4.4069	− 1.9110		− 0.2214	0.9585		4.0379
*Coefficients*						
	Estimate		Std. Error	z-value		Pr (> ǀzǀ)
(Intercept)	− 6.35899		0.25403	− 25.033		< 2e-16 ***
Discipline neurology	− 0.41811		0.11671	− 3.582		0.00034 ***
Discipline orthopaedics	− 0.67105		0.26294	− 2.552		0.01071 *
Number of students	− 0.13848		0.04236	− 3.269		0.00108 **
Rate for females	1.59762		0.32229	4.957		7.16e-07 ***
–						
Signif. codes	0 ‘***’	0.001 ‘**’	0.01 ‘*’	0.05 ‘.’	0.1. ‘’	1
(Dispersion parameter for poisson family taken to be 1)						
Null deviance: 252.59 on 35 degrees of freedom						
Residual deviance: 157.63 on 31 degrees of freedom						
AIC: 296.76						
Number of Fisher Scoring iterations: 5

**Table 6b:** Poisson model for the mistake rate, adjusted for the discipline, as well as the number of students and the rate for females without outlier

Poisson model for the mistake rate adjusted for the discipline as well as the number of students and the rate for females without outlier						
*Deviance residuals*						
Min	1Q		Median	3Q		Max
− 3.7356	− 1.8825		− 0.2277	0.8120		4.2507
*Coefficients*						
	Estimate		Std. Error	z-value		Pr (> ǀzǀ)
(Intercept)	− 6.21178		0.26140	− 23.764		< 2e-16 ***
Discipline neurology	− 0.12632		0.13238	− 0.954		0.3400
Discipline orthopaedics	− 0.58338		0.27076	− 2.155		0.0312 *
Number of students	− 0.07894		0.04530	− 1.743		0.0814
Rate for females	0.29918		0.41405	0.723		0.4700
–						
Signif. codes	0 ‘***’	0.001 ‘**’	0.01 ‘*’	0.05 ‘.’	0.1. ‘’	1
(Dispersion parameter for poisson family taken to be 1)						
Null deviance: 169.61 on 34 degrees of freedom						
Residual deviance: 127.02 on 30 degrees of freedom						
AIC: 260.25						
Number of Fisher Scoring iterations: 5						

**RQ 1: Which types of student mistakes occur during bedside teaching lessons?**

To answer this question, we describe the prevalence of the types of student mistakes in our video data (cf. Table 7). In internal medicine, reproduction mistakes (42.2%) were most frequent. In neurology and orthopaedics, the type of mistake with the highest prevalence was wrong application of skills (42.3% and 26.0%, respectively). Furthermore, in orthopaedics, different types of mistakes were more evenly distributed than in the other two disciplines.

**Table 7:** Absolute and relative frequencies of the different types of student mistakes in bedside teaching in three medical disciplines

Type of mistake	Discipline							
	Internal medicine		Neurology		Orthopaedics		Overall	
	Quantity	Ratio (%)	Quantity	Ratio (%)	Quantity	Ratio (%)	Quantity	Ratio (%)
Reproduction mistake	84	42.2	31	25.2	7	14.0	122	32.8
Comprehension mistake	29	14.6	18	14.6	8	16.0	55	14.8
Application mistake regarding skills	47	23.6	52	42.3	13	26.0	112	30.1
Application mistake regarding knowledge	31	15.6	10	8.1	11	22.0	52	14.0
Analytic mistake	0	0.0	11	8.9	9	18.0	20	5.4
Other mistakes	6	3.0	0	0.0	1	2.0	7	1.9
Mistakes related to misunderstandings/misinterpretations	2	1.0	1	0.8	1	2.0	4	1.1

**RQ 2: How do clinical teachers react to student mistakes in bedside teaching lessons?**

Table 8 shows the prevalence of the different types of teacher reactions to the mistakes made by medical students for the disciplines internal medicine, neurology and orthopaedics.

**Table 8:** Types of clinical teacher reactions: absolute and relative frequencies of the different qualities of teacher reactions to student mistakes

Teacher reactions to mistakes		Discipline							
		Internal medicine		Neurology		Orthopaedics		Overall	
		Quantity	Ratio (%)	Quantity	Ratio (%)	Quantity	Ratio (%)	Quantity	Ratio (%)
Feedback	Explicit rejection	91	45.7	103	83.7	37	74.0	231	62.1
	Further inquiry	108	54.3	20	16.3	13	26.0	141	37.9
Elaboration	Low	83	41.7	56	45.5	28	56.0	167	44.9
	High	116	58.3	67	54.5	22	44.0	205	55.1
Time for correction	Yes	105	52.8	45	36.6	18	36.0	168	45.2
	No	94	47.2	78	63.4	32	64.0	204	54.8

First, we look at the categorical scheme *feedback*, comprising *explicit rejection* and *further inquiry*. As shown in Table 8, the clinical teachers more often explicitly rejected a student mistake (62.1%) when compared to allowing further inquiry, which includes leaving false statements or examination techniques uncorrected at first. This trend of direct rejection is most apparent in neurology (83.7%) and orthopaedics (74.0%), whereas in internal medicine, teachers reacted to 54.3% of the mistakes with further inquiry instead of rejection. The differences between internal medicine and neurology (*p* = 0.001) as well as orthopaedics (*p* = 0.006) are statistically significant.

Regarding the *level of elaboration*, the overall result was an overweight of highly elaborated reactions to mistakes compared to reactions with low elaboration (55.1% vs. 44.9%). Nevertheless, in the three medical disciplines, we observed a more differentiated picture. In internal medicine, this general trend was even more pronounced, and the teachers reacted most often with a high level of elaboration (58.3%), i.e. by providing time to thoroughly discuss a mistake. This trend was also apparent in neurology but less pronounced with 54.5%. In orthopaedics, reactions with high levels of elaboration were less observed (44%). In this discipline teachers had the tendency to react with low elaboration (56.0%).

A further difference emerged in the field of *time allotted for the correction of the mistake.* Here, physicians from orthopaedics and neurology more often gave their students time to correct their mistakes (64.0% and 63.4%, respectively), whereas their colleagues from internal medicine allowed time for correction in only 47.2% of the cases.

For investigating the effect of the different disciplines to the different teacher reactions we proposed a generalized mixed model, respecting the dependency of lecturers. This was important as the same lecturers held several seminars.

### Generalized mixed models

For the generalized mixed model we proposed a random intercept, respecting the dependency of the covariate individual clinical teachers. This allowed to incorporate the subject-specific dependence structure into the model.

Feedback (cf. Table 9)

**Table 9:** Generalized mixed model results: Feedback

Generalized linear mixed model fit by maximum likelihood (Laplace Approximation) [glmerMod]						
AIC	BIC		logLik	Deviance		df.resid
436.5	459.8		− 212.2	424.5		355
*Scaled residuals*						
Min	1Q		Median	3Q		Max
− 1.7705	− 0.5929		− 0.4196	0.7196		2.9705
*Random effects*						
Name	Variance		Std. Dev			
(Intercept)	1.897		1.377			
Number of obs: 361						
*Fixed effects*						
	Estimate		Std. Error	z-value		Pr (> ǀzǀ)
(Intercept)	− 0.35779		0.38618	− 0.926		0.35419
Application Mistakes regarding Knowledge	− 0.48443		0.41547	− 1.166		0.24362
Analytic mistakes	− 0.19017		0.63403	− 0.300		0.76423
Reproduction mistakes	− 0.90869		0.33727	− 2.694		0.00705 **
Comprehension mistakes	− 0.02088		0.39388	− 0.053		0.95773
–						
Signif. codes	0 ‘***’	0.001 ‘**’	0.01 ‘*’	0.05 ‘.’	0.1. ‘’	1
*Correlation of fixed effects*						
	(Intr)		Application Mistake regarding Knowledge	Analytic Mistake		Reproduction Mistake
Application Mistake regarding Knowledge	− 0.355					
Analytic Mistake	− 0.225		0.198			
Reproduction Mistake	− 0.412		0.445	0.208		
Comprehension Mistake	− 0.367		0.347	0.214		0.440

In Table 9 we can see that the effect of a reproduction mistake had a significant influence to the outcome *feedback* compared to its reference category application mistake. In case of making a reproduction mistake compared to an application mistake, the odds ratio for an explicit rejection decreased by a multiplicative effect of exp(− 0.91) = 0.40. Elaboration (cf. Table 10).

**Table 10:** Generalized mixed model results: Elaboration

Generalized linear mixed model fit by maximum likelihood (Laplace Approximation) [glmerMod]						
AIC	BIC		logLik	Deviance		df.resid
491.3	514.7		− 239.7	479.3		355
*Scaled residuals*						
Min	1Q		Median	3Q		Max
− 2.6919	− 0.9212		0.5825	0.8307		1.3900
*Random effects*						
Name	Variance		Std. Dev			
(Intercept)	0.2007		0.448			
Number of obs: 361						
*Fixed effects*						
	Estimate		Std. Error	z-value		Pr (> ǀzǀ)
(Intercept)	0.3300		0.2279	1.448		0.1477
Application Mistakes regarding Knowledge	− 0.1170		0.3585	− 0.326		0.7442
Analytic Mistakes	1.4258		0.6522	2.186		0.0288 *
Reproduction Mistakes	− 0.6787		0.2851	− 2.381		0.0173 *
Comprehension Mistakes	0.3153		0.3550	0.888		0.3745
–						
Signif. codes	0 ‘***’	0.001 ‘**’	0.01 ‘*’	0.05 ‘.’	0.1. ‘’	1
*Correlation of fixed effects*						
	(Intr)		Application Mistake regarding Knowledge	Analytic Mistake		Reproduction Mistake
Application Mistake regarding Knowledge	− 0.515					
Analytic Mistake	− 0.270		0.178			
Reproduction Mistake	− 0.632		0.424	0.153		
Comprehension Mistake	− 0.503		0.323	0.180		0.412

With regard to low or high elaboration, analytic and reproduction mistakes had a significant influence compared to an application mistake. Making an analytic mistake compared to an application mistake, the odds ratio for a high elaboration increased by a multiplicative factor of exp(1.4) = 4.2. In case of reproduction mistakes with reference to application mistakes, the odds ratio for a high elaboration decreased by a multiplicative effect of exp(− 0.68) = 0.51.

Time for correction (cf. Table 11)

**Table 11:** Generalized mixed model results: Time for correction

Generalized linear mixed model fit by maximum likelihood (Laplace Approximation) [glmerMod]						
AIC	BIC		logLik	Deviance		df.resid
487.6	510.9		− 237.8	475.6		355
*Scaled residuals*						
Min	1Q		Median	3Q		Max
-2.5085	− 0.9922		0.5090	0.8607		1.6898
*Random effects*						
Name	Variance		Std. Dev			
(Intercept)	0.334		0.5779			
Number of obs: 361						
*Fixed effects*						
	Estimate		Std. Error	z-value		Pr (> ǀzǀ)
(Intercept)	0.4567		0.2531	1.805		0.0711
Application mistakes regarding knowledge	− 0.2278		0.3697	− 0.616		0.5378
Analytic mistakes	0.7528		0.6276	1.199		0.2303
Reproduction mistakes	-0.6087		0.2910	− 2.091		0.0365 *
Comprehension mistakes	-0.6448		0.3533	− 1.825		0.0680
–						
Signif. codes	0 ‘***’	0.001 ‘**’	0.01 ‘*’	0.05 ‘.’	0.1. ‘’	1
Correlation of fixed effects						
	(Intr)		Application mistake regarding knowledge	Analytic mistake		Reproduction mistake
Application mistake regarding knowledge	− 0.503					
Analytic mistake	− 0.279		0.194			
Reproduction mistake	− 0.622		0.452	0.231		
Comprehension mistake	− 0.502		0.350	0.204		0.450

Also the investigation whether the lecturers allowed time for correction or not showed a significant effect of reproduction mistakes compared to an application mistake. A reproduction mistake compared to an application mistake reduced the odds ratio for allowing time for correction was exp(− 0.61) = 0.54.

### Mixed models

The mixed models in Tables 12, 13, 14 allowed to incorporate the type of student mistake as well as the discipline for the outcomes feedback, elaboration and time for correction.

**Table 12:** Mixed model results: type of student mistake and discipline for the outcome feedback

Generalized linear mixed model fit by maximum likelihood (Laplace Approximation) [glmerMod]								
AIC	BIC		logLik	Deviance		df.resid		
420.3	451.4		− 202.1	404.3		353		
*Scaled residuals*								
Min	1Q		Median	3Q		Max		
− 1.8573	− 0.6268		− 0.4003	0.7247		3.5449		
*Random effects*								
Name	Variance		Std. Dev					
(Intercept)	0.416		0.645					
Number of obs: 361								
*Fixed effects*								
	Estimate		Std. Error	z-value		Pr (> ǀzǀ)		
(Intercept)	0.85269		0.34954	2.439		0.014710 *		
Application mistakes regarding knowledge	− 0.59424		0.41299	− 1.439		0.150193		
Analytic Mistakes	0.01992		0.61978	0.019		0.984612		
Reproduction mistakes	− 1.05795		0.33653	− 3.144		0.001668 **		
Comprehension mistakes	− 0.13734		0.39610	− 0.347		0.728802		
Discipline neurology	− 2.33726		0.49422	− 4.729		2.25e-06 ***		
discipline orthopaedics	− 2.01657		0.60570	− 3.329		0.000871 ***		
–								
Signif. Codes	0 ‘***’	0.001 ‘**’	0.01 ‘*’	0.05 ‘.’	0.1. ‘’	1		1
*Correlation of fixed effects*								
	(Intr)		Application Mistake regarding Knowledge	Analytic Mistake		Reproduction Mistake	Comprehension Mistake	Discipline Neurology
Application Mistake regarding Knowledge	− 0.428							
Analytic Mistake	− 0.149		0.193					
Reproduction Mistake	− 0.571		0.451	0.192				
Comprehension Mistake	− 0.412		0.354	0.208		0.439		
Discipline Neurology	− 0.554		0.108	− 0.086		0.176	0.050	
Discipline Orthopaedics	− 0.428		− 0.028	− 0.183		0.109	− 0.030	0.363

**Table 13:** Mixed model results: type of student mistake and discipline for the outcome elaboration

Generalized linear mixed model fit by maximum likelihood (Laplace Approximation) [glmerMod]						
AIC	BIC	logLik	Deviance	df.resid		
489.0	520.1	− 236.5	473.0	353		
*Scaled residuals*						
Min	1Q	Median	3Q	Max		
− 2.2990	− 0.9849	0.6248	0.8977	1.7339		
*Random effects*						
Name	Variance	Std. Dev				
(Intercept)	2.842e− 14	1.686e-07				
Number of obs: 361						
*Fixed effects*						
	Estimate	Std. Error	z-value	Pr (> ǀzǀ)		
(Intercept)	0.6455	0.2395	2.695	0.00703 **		
Application Mistakes regarding Knowledge	-0.1160	0.3511	− 0.330	0.74112		
Analytic Mistakes	1.4491	0.6133	2.363	0.01815 *		
Reproduction mistakes	− 0.7504	0.2771	− 2.708	0.00677 **		
Comprehension mistakes	0.2953	0.3485	0.847	0.39681		
Discipline neurology	− 0.4296	0.2510	− 1.712	0.08693		
Discipline orthopaedics	− 0.9959	0.3604	− 2.763	0.00573 **		
–-						
Signif. codes	0 ‘***’	0.001 ‘**’	0.01 ‘*’	0.05 ‘.’	0.1. ‘’	1
*Correlation of fixed effects*						
	(Intr)	Application mistake regarding knowledge	Analytic mistake	Reproduction mistake	Comprehension mistake	Discipline neurology
Application Mistake regarding Knowledge	− 0.528					
Analytic mistake	− 0.177	0.184				
Reproduction mistake	− 0.720	0.420	0.183			
Comprehension mistake	− 0.488	0.325	0.181	0.405		
Discipline neurology	− 0.562	0.171	− 0.070	0.246	0.081	
Discipline orthopaedics	− 0.337	− 0.032	− 0.242	0.164	− 0.018	0.314
Optimizer (Nelder_Mead) convergence code: 0 (OK)						
Boundary (singular) fit: see help (‘isSingular’)

**Table 14:** Mixed model results: type of student mistake and discipline for the outcome time for correction

Generalized linear mixed model fit by maximum likelihood (Laplace Approximation) [glmerMod]						
AIC	BIC	logLik	Deviance	df.resid		
489.8	520.9	− 236.9	473.8	353		
*Scaled residuals*						
Min	1Q	Median	3Q	Max		
− 2.4683	− 0.9758	0.5123	0.8654	1.6918		
*Random effects*						
Name	Variance	Std. Dev				
(Intercept)	0.2484	0.4984				
Number of obs: 361						
*Fixed effects*						
	Estimate	Std. Error	z-value	Pr (> ǀzǀ)		
(Intercept)	0.2386	0.2970	0.803	0.4217		
Application Mistakes regarding Knowledge	− 0.2270	0.3693	− 0.615	0.5388		
Analytic Mistakes	0.6321	0.6295	1.004	0.3154		
Reproduction Mistakes	− 0.5820	0.2892	− 2.012	0.0442 *		
Comprehension Mistakes	− 0.6444	0.3522	− 1.829	0.0673		
Discipline Neurology	0.4233	0.3759	1.126	0.2601		
Discipline Orthopaedics	0.5324	0.4639	1.148	0.2511		
–						
Signif. codes	0 ‘***’	0.001 ‘**’	0.01 ‘*’	0.05 ‘.’	0.1. ‘’	1
*Correlation of fixed effects*						
	(Intr)	Application mistake regarding knowledge	Analytic mistake	Reproduction mistake	Comprehension mistake	Discipline neurology
Application mistake regarding knowledge	− 0.440					
Analytic mistake	− 0.169	0.196				
Reproduction mistake	− 0.574	0.447	0.221			
Comprehension mistake	− 0.434	0.352	0.206	0.448		
Discipline neurology	− 0.519	0.083	− 0.077	0.083	0.036	
Discipline orthopaedics	− 0.386	− 0.059	− 0.158	0.063	− 0.027	0.319

**RQ 3: Do clinical teachers use different strategies to address different types of student mistakes?**

To answer the third research question it was investigated whether clinical teachers responded differently to different types of students’ mistakes. We use stacked bar charts to illustrate our results.

**Fig. 4:**
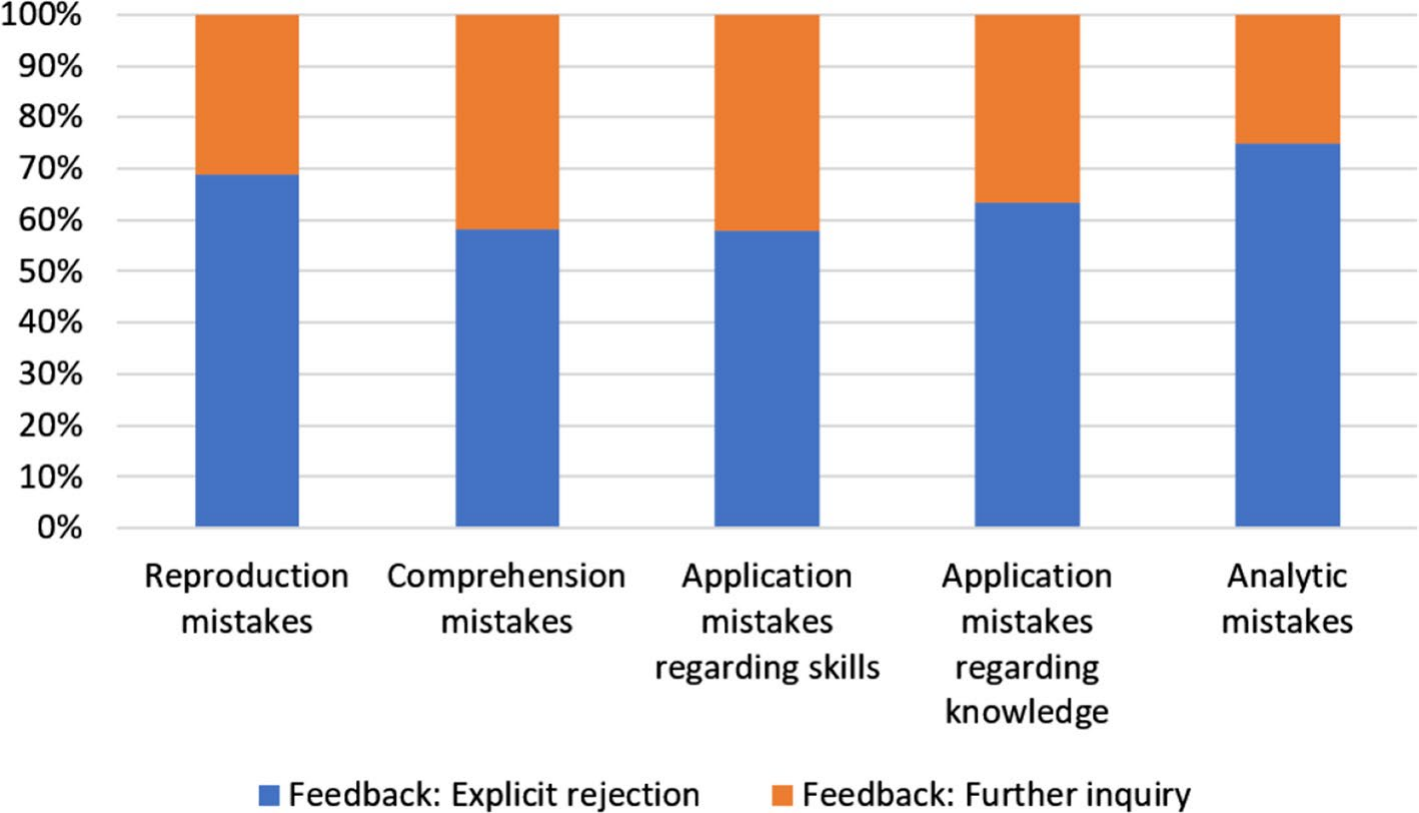
Stacked bar chart depicting the teachers’ feedback to the different types of student mistakes

In Fig. 4 we can see the feedback of clinical lecturers to the different types of mistakes. For each type of mistake there was more often an explicit rejection observed compared to a further inquiry. The type of mistake associated with the highest amount of cognitive complexity, i.e. analytic mistake, was associated with the highest degree of open rejection.

**Fig. 5:**
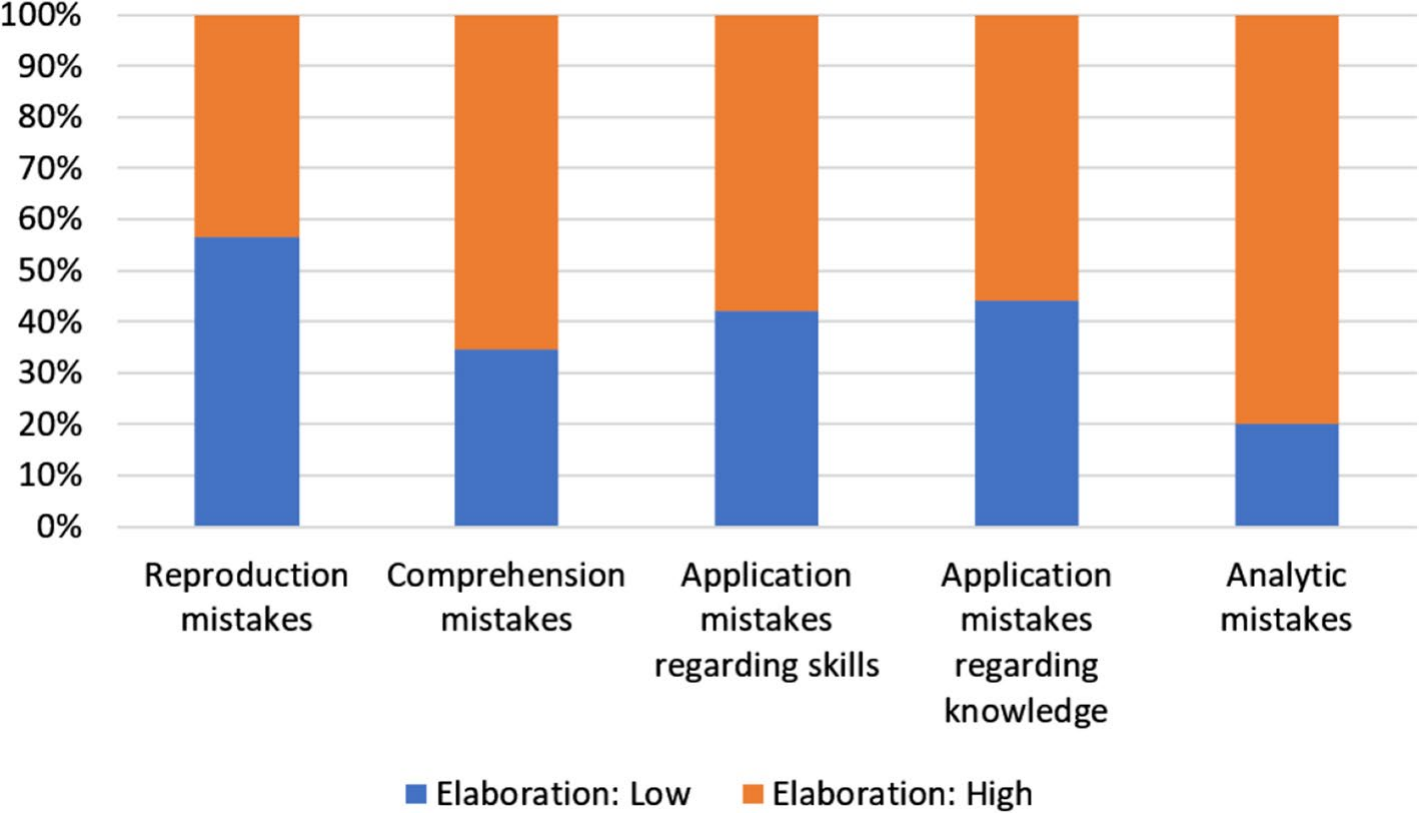
Stacked bar chart depicting the teachers’ levels of elaboration reacting to the different types of student mistakes

**Fig. 6:**
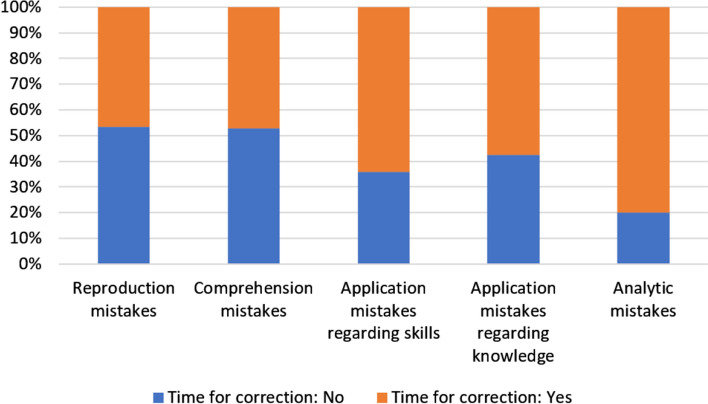
Stacked bar chart depicting the time allocation for the correction of the different types of mistakes

Regarding the *level of elaboration* (Fig. 5), the clinical teachers reacted with a higher level of elaboration to application mistakes (both skills and knowledge, 58.0% and 55.8%, respectively), information retrieval mistakes (80.0%) and comprehension mistakes (65.5%) compared to reproduction mistakes (43.4%). After correcting for multiple testing, only the difference in central tendency between the reproduction mistakes and information retrieval mistakes was significant (*p* = 0.023).The data in Fig. 6 show that clinical teachers least frequently allowed time for correction of mistakes representing the lowest levels of cognitive complexity, i.e. reproduction and comprehension mistakes. Only after 46.9% of such mistakes, the students were given time for reflection/correction. In cases where students wrongly applied their knowledge or skills, the clinical teachers allowed time for correction of these mistakes in 62.2% of the cases observed. Finally, in 80.0% of the analytic mistakes made by the students, the teachers allowed time for correction, which was the highest value in our study.

## Discussion

In this study, we report a video-analytic study on bedside teaching lessons from *internal medicine*, *neurology* and *orthopaedics*. Based on our analyses, three different research questions were addressed: (1) which types of student mistakes can be observed in curricular bedside teaching sessions, (2) how do clinical teachers react to these mistakes and (3) do they use different strategies to address different types of mistakes? In the following section, we will focus on the main results of our study and put these into perspective regarding research-related, practical and methodological considerations.

### Frequencies of the different types of student mistakes

Researchers have argued that the students’ knowledge-related misconceptions can be detrimental for further learning and that it is a great challenge for medical educators to detect the students’ misconceptions and address them didactically (Olde Bekkink et al., 2016). From a learning theoretical perspective (e.g., Tulis et al., 2016), each student mistake presents an opportunity for clinical teachers to investigate whether other students have similar misconceptions. In line with this, these misconceptions can be purposefully addressed by discussing them in small groups. This also applies to students’ clinical skills, as shown in our results. Our study outcomes indicate that bedside teaching provides a very promising context here: we observed a high frequency of students verbalising erroneous knowledge about medical subject matter or making mistakes on the level of practical clinical skills.

In existing descriptions and empirical studies on bedside teaching, the opportunity for students to learn from their (knowledge and skills-related) mistakes is sometimes hinted about or mentioned (e.g. Peters & Cate, 2014; Ramani, 2003) but has so far not yet been discussed in conceptual or empirical studies. In Ramani, 2003 article *twelve tips to improve bedside teaching*, which is still frequently cited in the medical education literature, the notion of using student mistakes as starting points for teaching and learning plays only a minor role. Through tips, such as ‘gentle corrections can be made when needed’ or ‘admitting one’s own lack of knowledge might set the tone for trainees to admit their limitations and engender a willingness to ask questions’, Ramani, 2003 only vaguely hint towards the potential of learning from mistakes in bedside teaching. Moreover, a notion is advanced about the clinical teachers’ main responsibility to correct student mistakes. In our view, this notion is not in line with the contemporary insights of educational researchers about how (clinical) teachers can foster learning from mistakes, e.g. through guiding students to recognise, understand and correct their mistakes themselves (e.g. Grassinger et al., 2018).

Overall, across the clinical domains, we observed an average of 10 student mistakes per bedside teaching lesson. This means that in an average bedside encounter, a clinical teacher gets about 10 opportunities to assist students in becoming aware of and learning from misconceptions in their clinical knowledge and skills. This result underscores the relevance of taking learning from mistakes into account as an important yet rarely considered aspect of bedside teaching. When using student mistakes as a starting point for learning, clinical teachers can focus on the subject matter, which is relevant for their students simply because they get to work with the limitations in the students’ knowledge and skills.

In the present study, close to one-third of all mistakes observed were reproduction-related, followed by mistakes relating to the wrong application of skills (approximately 30%). Considering that bedside teaching is one of the most practical teaching methods in undergraduate medical education, the proportion of application-related errors seems to be rather small. Nevertheless, the results indicated two functions of bedside teaching that have, until now, not been studied, i.e. *to address the students’ misconceptions on the level of basic knowledge and its application to patients* and *to improve examination skills by correcting deficits in the application of these skills*. Thus, clinical teachers should be more aware of bedside teaching offering opportunities to help students in correcting their misconceptions both, on the levels of knowledge and skills.

### Domain effects

Regarding different types of mistakes and their frequencies in different clinical domains, we found only slight disparities between the three medical disciplines.

One difference was observed in mistakes that occur when applying existing skills in new situations. As shown in Table 1, the medical students made most of the mistakes in neurology when applying skills (42.3%) (internal medicine and orthopaedics: 23.6% and 26.0%, respectively), possibly indicating that neurological teachers give great importance to the application of skills and thus provide more possibilities for such exercises during the lessons.

Another difference between disciplines was observed in the strongest trend towards open rejection of student mistakes being apparent in neurology. Bedside teaching differs slightly in the three disciplines, e.g. with a stronger focus on practising clinical skills in neurology. Thus, we conclude that lecturers are more inclined to openly reject student mistakes on the level of practical clinical skills than on the level of knowledge. However, if we look more closely at teachers’ feedback to application mistakes (skills and knowledge) across domains, we see a slightly higher rate of explicit rejection when teachers are reacting to application mistakes regarding knowledge rather than skills.

Theorisation and research on effective feedback confirm that open rejection is associated with negative emotional reactions of feedback recipients (Lefroy et al., 2015). Hence, it seems worthwhile for medical educators to educate clinical (bedside) teachers about better ways to react to student mistakes.

Furthermore, we observed that mistakes were made in the areas of analysis, evaluation and creation (information retrieval mistakes: 5.4%). Outliers in information retrieval mistakes in orthopaedics (18.0%) were present. However, this may be explained by a relatively low total number of mistakes observed in orthopaedics (50 versus 199 in internal medicine). One possible reason for this surprisingly low total number of recorded mistakes is that students in orthopaedic bedside teaching rounds often did not perform a clinical examination on the patient because of the large number of post-operative patients in this discipline. Another reason could be the larger average number of students and the higher proportion of talks by the teacher, both of which lead to students only participating, if at all, when they know the right answer to a question.

### Teacher reactions to student mistakes

To which degree do clinical teachers manage to tap into the didactic potential associated with student mistakes, i.e. to make these useful as starting points for learning processes (Bauer, 2008; Edmondson, 1999). In this respect, our results were mixed. Instances of teacher feedback on student mistakes more often had the character of *explicit rejection* as compared to *further inquiry* (with an approximate 3:1 ratio)*.* In many situations, clinical teachers also allowed *no time for correction of the mistake*. These two results hint towards teachers failing to give students opportunities, at first, to recognise that something they had contributed was incorrect and, second, to reflect upon and correct their answer (Mindnich et al., 2008). Existing research confirms that the more individuals see error situations as starting points for learning, the more likely they are to engage in learning activities, such as communication about the error (Bauer, 2008). We argue that by not reacting with *explicit rejection*, bedside teachers can act as role models for students in that they treat their mistakes not just as unfortunate instances that need correction but as natural and valuable elements of learning processes. When discussing mistakes that occur in bedside teaching, it is important to keep in mind that not only students and teachers are involved, but mistakes can also occur in the presence of the patient. These mistakes not only hold the potential for students to embarrass themselves in front of a patient, but can also affect the doctor–patient relationship (Williams et al., 2008). It is likely that doctors are aware of this situation and adjust their response in front of the patient. Further research on how patients perceive mistakes in bedside teaching and on how teachers react to changes in the presence and absence of patients is needed.

Finally, we investigated to which degree clinical teachers adapt their reactions to different types of student mistakes. In this respect, we found only weak evidence for adaptive teacher behaviours. We argue that with different degrees of complexity of cognitive processes underlying mistakes (cf. previous section), it could be didactically reasonable for clinical teachers to adapt their reactions to student mistakes, e.g. to allow for *further inquiry* regarding the mistake, to initiate discussion regarding the underlying assumptions (category: *level of elaboration*) and to give students *time for correction of the mistake.*

This confirms our point that medical educators should strive to improve how they handle student mistakes in interactive forms of medical education. However, the learning effect of mistakes is hard to measure. Further experiments/studies with more complex designs would be needed.

### Limitations

Some of our analyses and results focus on differences between three medical disciplines. However, these can only be regarded as explorative, as for such analyses, our sample was too small. Because of the very limited number of studies on bedside teaching and the resulting lack of (notably international) possibilities of comparison, the promising results of our study should be validated via further video studies on bedside teaching.

Also, we could reject the hypothesis that our camera would potentially disturb clinical teachers and students: We found that the students did not evaluate the seminars in which our camera was present as significantly different from the other seminars (*p* = 0.572). With an average of 1.7 points on the Likert scale (1–6), the lecturers’ assessment was between ‘1 = not at all’ and ‘2 = slightly’ (Vagias, 2006) as to how much influence the camera had on the course of the seminar. Interestingly, the lecturers evaluated the influence on the behaviour of the students with 2.0 (‘slightly’) on average, thus indicating a greater influence on their behaviour than on the course of the seminar. These findings reflect the assessments of other studies in medical education (Groener et al., 2015).

## Conclusions

Our results provide an insight into mistake-related student–teacher interactions during bedside teaching sessions. Hence, we argue that they contribute some insights for advancing clinical teaching—as well as for the professional development of clinical teachers. Some suggestions for physicians during bedside teaching can be deducted from our findings: When seeking to further tap into the learning potential of student mistakes, teachers should adapt their reactions to the individual mistakes. Depending on the type of mistake, they should purposefully vary the quality of feedback they give, the level of elaboration and the time they allow for the correction of the mistake. At the risk of stating the obvious, we would like to emphasise that this learning potential can only be tapped by the teacher if they are present at the bedside.

## Data Availability

The datasets generated and analysed during the present study are available from the corresponding author upon reasonable request.

## Footnotes

1 Also cf. matrix of the classification, (Brand et al., 2005).

2 Please note that Anderson et al.,; 2001 differentiated two further levels, *evaluating* and *creating*, which were not applicable in the context of our study and hence are not mentioned here.

3 Code of mistake.

4 Besides, this category may show in a teacher posing other questions or explicating more to help the student comprehend (without stating that the first answer was wrong).
